# Chemosensitizing and nephroprotective effect of resveratrol in cisplatin –treated animals

**DOI:** 10.1186/s12935-014-0152-2

**Published:** 2015-02-04

**Authors:** Abdel-Moneim M Osman, Saud A Telity, Zoheir A Damanhouri, Sameer E Al-Harthy, Huda M Al-Kreathy, Wafaa S Ramadan, Mohamed F Elshal, Lateef M Khan, Fatemah Kamel

**Affiliations:** Pharmacology Department, Faculty of Medicine, King Abdulaziz University, P.O. box 80205, Jeddah, Saudi Arabia; Pharmacology unit, National Cancer Institute, Cairo University, Cairo, 11796 Egypt; Anatomy Department, Faculty of Medicine, King Abdul Aziz University, P.O. box 80205, Jeddah, Saudi Arabia; Department of biochemistry, Faculty of science, king abdulaziz University, P.O. Box 80203, Jeddah, Saudi Arabia; Molecular Biology and Genetic engineering and Biotechnology Department, Minoufia Universitym Minoufia, Minoufia, Egypt

**Keywords:** Cisplatin, Resveratrol, Nephroprotection

## Abstract

**Background:**

Cisplatin (CIS) is one of the most effective anticancer drug used in the treatment of several solid tumors .Its use is limited by its nephrotoxicity. The present study was designed to assess the role of a natural product resveratrol (RSVL) on sensitization of mammary carcinoma (Ehrlich ascites carcinoma) to the action of CIS and the possible protective effect against CIS-induced nephrotoxicity in rats.

**Methods:**

The percent survival of female tumor bearing mice was used for determination the cytotoxic activity of CIS in the presence or the absence of RSVL. Uptake and cell cycle effect, serum creatinine (CREA), blood urea nitrogen (BUN), Reduced Glutathione (GSH) and histopatholgical examination of kidney tissues after CIS and/or RSVL therapy were also investigated.

**Results:**

RSVL increased the intracellular level of CIS in EAC cells and there was a strong correlation between the high cellular level of CIS and its cytotoxicity. CIS at a dose level of 5 mg/kg increased the mean survival time of female tumor bearing mice to 25 days compared with 17 days for tumor-bearing control mice. Administration of RSVL at a dose level of 25 mg/kg simultaneously with CIS increased the mean survival time to 48 days with 60% survival of the tumor-bearing animals. Cell cycle analysis of tumor cells showed that CIS treatment decreases the proliferation index of tumor cells while in presence of RSVL there was more significant inhibitions. Also, CIS treatment caused increase in level of creatinine and blood urea with significant decrease in the GSH level. While, in the presence of RSVL, level of creatinine and blood urea restored to control level.

**Conclusion:**

This study suggests that RSVL could increase the cytotoxic activity of CIS and protect against its nephrotoxicity.

## Introduction

Cisplatin is commonly used cytotoxic drug in the management of several human solid tumors such as neck and head, testis, ovary, bladder, colorectal and breast cancers. Whilst early good responses to drug after the first cycles of the treatment, CIS usually acquired resistance and severe toxicity including renal, neuro, oto, gastro toxicity as well as myelosuppression [1]. It has been reported that reactive oxygen species (ROS) play an important role in CIS-triggered acute renal failure [2], and also trigger depletion of glutathione which play a role in nephrotoxicity [3,4]. The alternative strategy is to develop co-therapies that reduce renal damage by counteracting CIS effects on the kidney. In an attempt to minimize the serious side effects of anticancer drugs ,a variety of approaches have been investigated. A natural product like resveratrol (RSVL) which have an additive cytotoxic activity has been tried with different type of anti-cancer drugs against different cancer cells [5]. Resveratrol is one of promising dietary chemopreventive phytochemicals chemotherapeutic potential. It has wide-spectrum beneficial effect, such as anti-infective, anti-oxidant and cardioprotective function [6]. In light of these findings the goal of this study is to examine the possible effect of RSVL treatment in enhancing the antitumor activity of CIS by examining CIS antitumor activity and cell cycle distribution. In addition, examining the possible renal protective effect of RSVL against CIS triggered nephrotoxicity.

## Materials and methods

### Drugs and chemicals

Cisplatin (CIS) and resveratrol (RSVL) were purchased from Sigma Aldrich Co. (Saint Louis, Missouri, USA). The stock solution of both drugs (dissolved in phosphate buffer saline (PBS) and preserved at −20°C). The solutions were diluted in normal saline immediately before each experiment to the desired final concentration.

### Animals and tumor

Female Swiss albino mice (8 weeks of age, 20–22 g body weight) and Female Wistar albino rats (8–10 weeks of age, 180-200 g body weight) were obtained from King Fahd Medical Research center, King Abdulaziz University, Jeddah, Saudi Arabia. The animals were acclimatized for one week before each experiment. A commercial balanced diet and water *ad libitum* were provided throughout the experiment.

The Ehrlich ascites carcinoma cells (EAC) cells was acquired through the courtesy of National cancer institute (Cairo) and maintained in our laboratory by weekly I.P. transplantation of 2.5 × 10^6^ cells/mouse. This study was approved by the Institutional ethical committee of King Abdulaziz hospital.

### Evaluation of antitumor activity

The effect of RSVL on the antitumor activity of CIS against the growth of EAC was evaluated using the modified regimen of Donenko *et al.* [7]. Ehrlich ascites carcinoma cells were inoculated i.p. into forty Swiss albino mice (20–22 g) 2.5 × 10^6^ cells/mouse. Twenty four hours later, mice were equally divided into four groups. Group I injected with normal saline i.p. (0.2 ml/20 gm) and served as control group. Group II was administered with CIS (5 mg/kg i.p.) while group III received a single dose of RSVL (25 mg/kg i.p.). Group IV received RSVL (25 mg/kg i.p.) simultaneously with CIS (5 mg/kg i.p.).

Average survival days of mice and long term survivors are defined as the mice survived to the end of the experiment (45 days) with no apparent tumor.

### Measurement of Cisplatin cellular uptake

CIS cellular accumulation assessment in Ehrlich ascites cells was performed using ICP-Optical Emission Spectrometer (Optima 7000 DV ICP-OES perkinelmer, Inc. Waltham, USA). EAC cells were inoculated i.p. into thirty six Swiss albino mice (20–22 g) 10 × 10^6^ cells/mouse. Twenty four hours later, mice were divided into six groups (six mice each). Groups 1,2 and 3 injected with CIS (7.5 mg/kg, i.p.). while, groups 4, 5 and 6 injected with RSVL (25 mg/kg, i.p.) simultaneously with CIS (7.5 mg/kg, i.p.). Animals were sacrificed by cervical dislocation at 3, 24 and 48 hours after treatment. Cell withdrawn from peritoneal cavity and washed twice with PBS and then suspended in 1 ml of normal salain. Then, centrifuged at 5000 rpm for 20 minute and washed once by PBS. Cells were counted and 2 ml of 1% nitric acid were added to the samples and kept in oven at 70 C for 24 hours. The volume of samples were completed to 5 ml with PBS then CIS concentration measured by ICP-Optical Emission Spectrometer (Optima 7000 DV ICP-OES PerkinElmer, Inc. Waltham, USA) at wavelengths 203 nm, 210 nm and 214 nm, respectively. Cell-uptake platinum was expressed as ppt of platinum per 1 × 10^6^ cells.

### Cell cycle analysis

Ehrlich ascites carcinoma cells were inoculated i.p. into sixty Swiss albino mice (20–22 g) 10 × 10^6^ cells/mouse and processed as mentioned above. Tumor cells were obtained at fixed times after CIS treatment. Cell cycle analysis was performed using flow cytometer (Becto Dicknson, BD, FACScalbur, USA according to the method of Smets et al. [8].

### Nephrotoxic effect of CIS in presence of RSVL

Fifty six female Wister rat were divided into four equal groups, each composed of fourteen animals each. Groups I received normal saline i.p. (0.5 ml/200 gm) and served as control group. Group II received RSVL (25 mg/kg body weight, i.p.). Group III received a single dose of CIS (7.5 mg/kg body weight, i.p.). Group IV received RSVL (25 mg/kg body weight, i.p.) and CIS (7.5 mg/kg body weight, i.p.) simultaneously. At the end of experiment period (48 hours), rat were anesthetized and blood samples were collected from 5 animals from each group from the ophthalmic artery in the orbital rim and rapidly centrifuged for Serum separation then stored at −80°C to evaluate serum creatinine (CREA) and blood urea nitrogen (BUN) which were determined using commercial kits (Siemens healthcare diagnostics ltd, USA) by Dimension Vista 1500 Intelligent Lab System (Siemens, USA) according to manufacturer instructions. kidney specimens from each groups (3 animals) were fixed in 10% formalin for light microscopic study. The residual kidney pieces (6 animals) was weighted and homogenized in 7 ml cold buffer (50 mM potassium phosphate, pH 7.5, 1 mM EDTA) per gram tissue by Homogenizer (Potters, German) then it was centrifuged at 4000 rpm for 15 minutes at 4°C. The supernatant was removed and stored in −80°C and used for the evaluation of reduced glutathione (GSH) by colorimetric method using biodiagnostic kit (Biodiagnositc, Egypt), according to the method of Beutler *et al.,* [9].

### Statistical analysis

Statistical analysis was performed using SPSS (Statistical package of social science, version 16). One way analysis of variance (ANOVA) followed by least significant difference (LSD) for post hoc analysis was used for multiple comparisons. Statistical significance was acceptable to a level of p ≤ 0.05.

## Results

### Survival of tumor bearing mice

**Figure 1:**
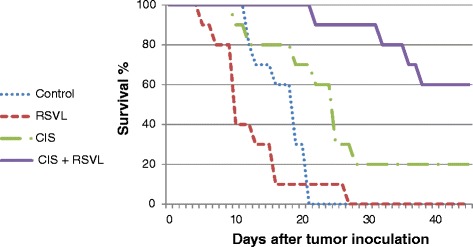
**Effects of CIS and/or RSVL treatment on the antitumor activity of CIS in mice bearing Ehrlich ascites carcinoma cell.**

Table 1 and Figure 1 show the effect of treatment with CIS and/or RSVL on the survival of tumor bearing mice. Tumor-bearing control mice showed mean survival time of 17 days, whereas ,administration of a single dose of CIS (5 mg/kg,i.p.) increased the mean survival time to 25 days, with 20% long term survivors. Simultaneous treatment with RSVL (25 mg/kg) significantly increased the mean survival time of tumor-bearing mice to 48 days with 60% survivors.

**Table 1:** **Effects of CIS and/or RSVL treatment on the therapeutic action of CIS in mice bearing Ehrlich ascites carcinoma cells**

**Treatment**	**Mean survival time(days)**	**45-day survivors**
**Control**	17 ± 3.74	0/10
**RSVL (25 mg/kg)**	12 ± 5.95^**b**^	0/10
**CIS (5 mg/kg)**	25 ± 12.13	2/10
**CIS (5 mg/kg) + RSVL (25 mg/kg) (supplied simultaneously)**	48 ± 15.03^**a,b**^	6/10

Each data represents the mean ± S.D. of ten mice.

^**a**^Significantly different from control at P-value < 0.05. ^**b**^Significantly different from CIS at P-value < 0.05.

### CIS level in tumor cells

**Figure 2:**
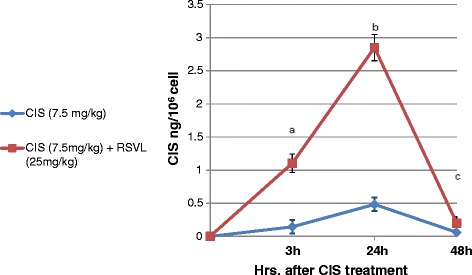
**Effect of RSVL treatment on the CIS cellular uptake in EAC cells.** (Blue Diamond) CIS was injected (7.5 mg/kg) in tumor-bearing mice, (Red square) CIS and RSVL (25 mg/kg) in simultaneous manner. Data represents the mean ± S.D. of six mice. ^**a**^Significantly different from Corresponding CIS after 3 hrs.of treatment at P > 0.05. ^**b**^significantly different from Corresponding CIS after 24 hrs.of treatment at P > 0.05. ^**c**^Significantly different from Corresponding CIS after 48 hrs.of treatment at P > 0.05.

Figure 2 show the cellular level of CIS in Ehrlich ascites cells after a single dose of CIS (7.5 mg/kg,i.p.) and/or RSVL (25 mg/kg,i.p.). All the time point tested showed that simultaneous RSVL treatment significantly increased the cellular uptake of CIS in the tumor cells with maximum level 24 hours after treatment (about 5.6 fold increase)

.

### Effect of CIS and /or RSVL treatment on cell cycle phase progression in Ehrlich cells

**Figure 3:**
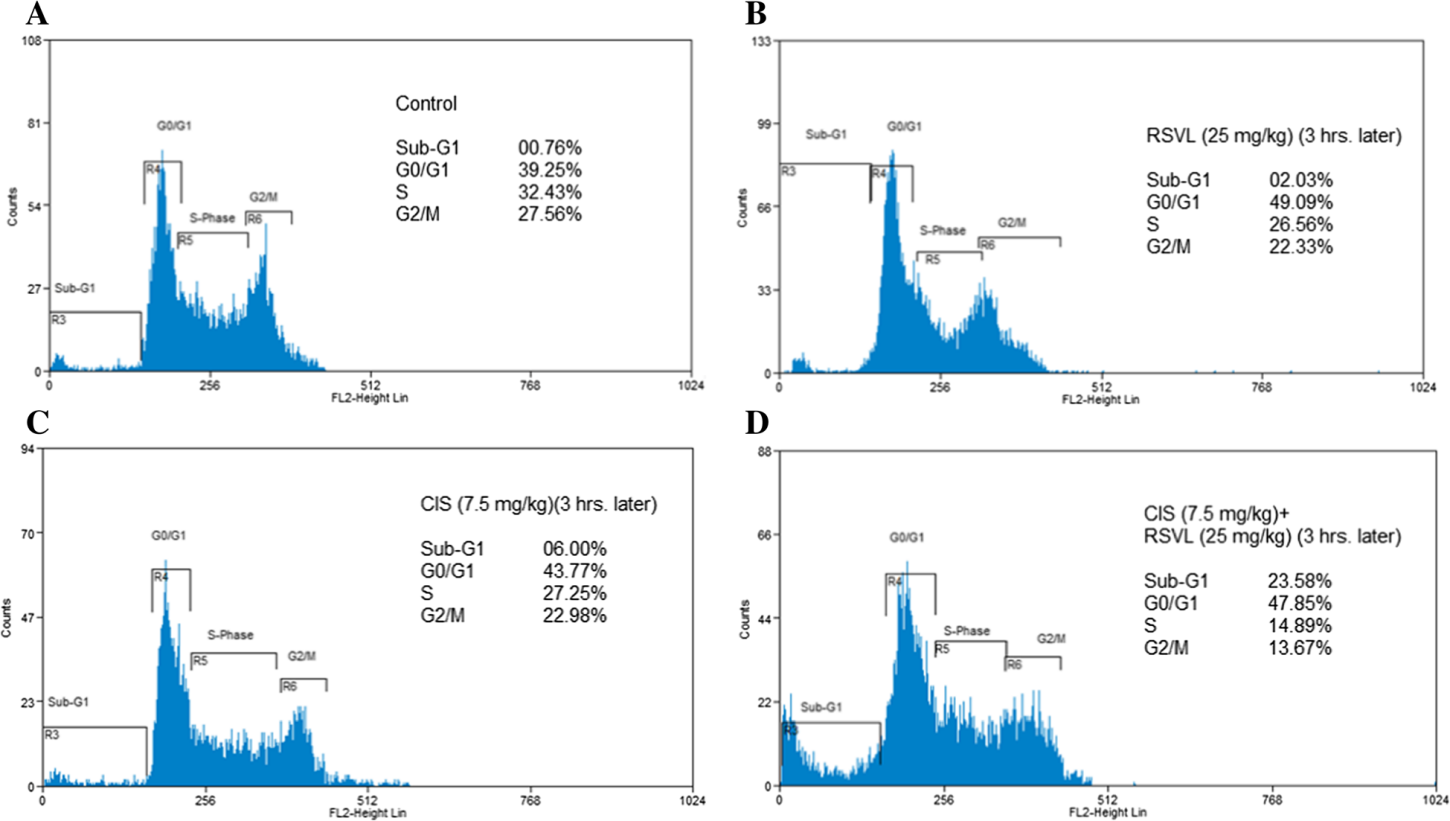
**Effect of CIS and/or RSVL treatment on cell cycle phase distribution of EAC cells.** Cell cycle distribution was analyzed after 3 hrs. of exposure to drugs by staining with propidium iodide. **(A)** Control), **(B)** Cells treated with RSVL (25 mg/kg). **(C)** Cells treated with CIS (7.5 mg/kg). **(D)** Cells treated with CIS (7.5 mg/kg) and RSVL (25 mg/kg).

CIS treatment (7.5 mg/kg) accumulated the dead cells in Sub-G_1_ phase by 6% after 3 hrs. of treatment, while combination treatment of CIS and RSVL showed more significantly accumulation of the dead cells in Sub-G_1_phase by 23.58% after 3 hrs. with maximum accumulation after 24 hrs. (data not shown). Moreover, there was more arrest in G_0_/G_1_ phase with maximum arrest after 3 hrs. by about 47.85% compared to control (Figure 3). Three, 24 and 48 hours after CIS treatment the proliferation index (S phase + G_2_/M phase) inhibited by about 16.38,38 and 38%, respectively (Table 2). Concomitant treatment with RSVL reduced the proliferation index significantly by about 52, 72 and 54%, respectively.

**Table 2:** **Effect of CIS and/or RSVL on proliferative activity of EAC cells**

	**Time after treatment**		
**Groups**	**3 hrs.**	**24 hrs.**	**48 hrs.**
**Control**	59.99 ± 1.38		
**RSVL (25 mg/kg)**	48.89^**a**^ ± 2.02	46.87^**a,b**^ ± 2.14	48.20^**a,b**^ ± 2.07
**CIS (7.5 mg/kg)**	50.23^**a**^ ± 4.73	36.93^**a**^ ± 3.05	37.20^**a**^ ± 1.96
**CIS (7.5 mg/kg) + RSVL (25 mg/kg)**	28.56^**a,b**^ ± 4.29	16.79^**a,b**^ ± 3.43	27.78^**a,b**^ ± 2.89

Data represents the mean ± S.D. of six mice. ^**a**^Significantly different from Control at P > 0.05. ^**b**^Significantly different from corresponding treatment of CIS at P > 0.05.

### Protective effect of RSVL against CIS-induced nephrotoxicity

**Figure 4:**
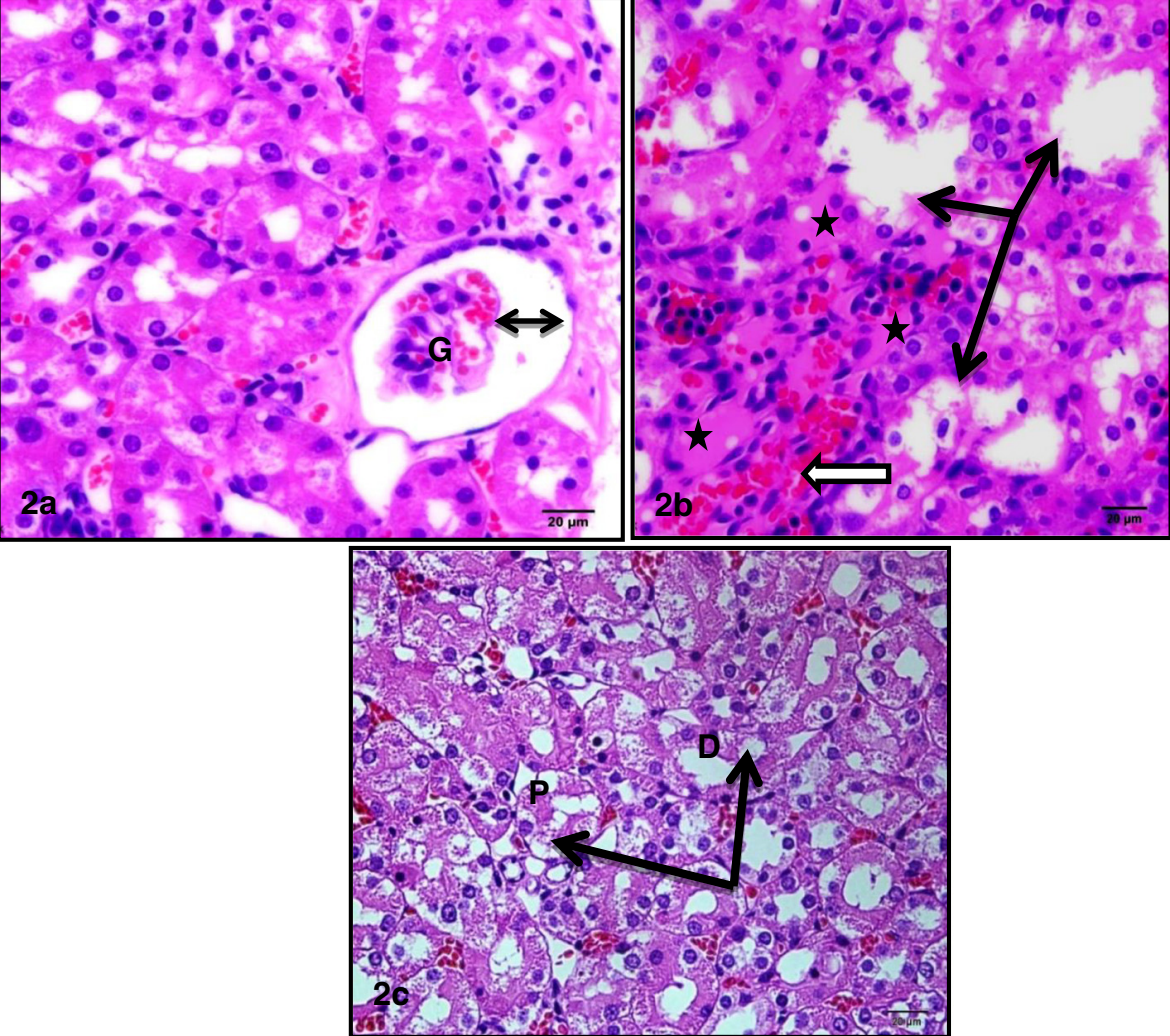
**Photomicrographs of sections of the kidney of a rat treated with CIS showing (2a) cortex with shrunken capillary tuft of the glomerulus(G) and widening of the subcapsular space (double head arrow). (2b)** deeper cortex with desquamated tubular epithelium (black arrows), deposition of homogenous material (casts) in their lumen (star) and intertubuluar congestion (white arrow). **(2c)** vacuolation of the cytoplasm (arrows) of both proximal (P) and distal (D) convoluted tubules.

**Figure 5:**
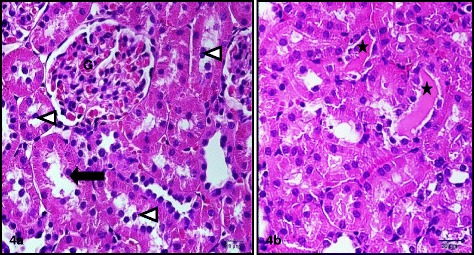
**Photomicrographs of sections of the kidney of a rat treated with RSVL and CIS simultaneously showing (4a) the cortex with normal glomerulus (G), dilated tubules (black arrow) and flaking of cells into tubular lumen (white triangles) (4b) deeper cortex revealing intratubular homogenous cast material (stars).**

**Figure 6:**
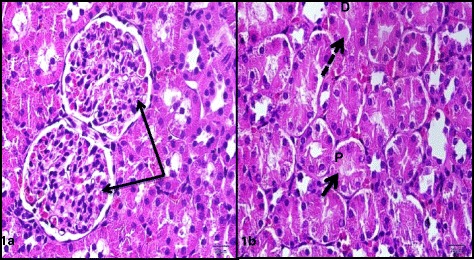
**Photomicrographs of sections of the kidney of a rat of the control group showing (1a) cortex with normal structured glomeruli (G) and subcapsular space (arrows).** (b) deeper cortex with proximal (P) and distal (D) convoluted tubules having cuboidal cells with eosinophilic cytoplasm and round basal nuclei (black arrow).

**Figure 7:**
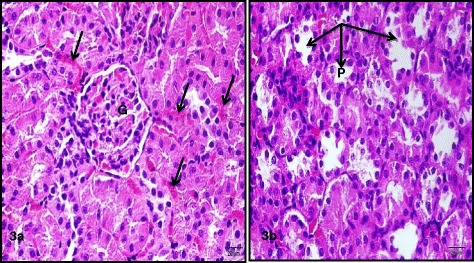
**Photomicrographs of sections of the kidney of a rat treated with RSVL showing (3a) the cortex with normal structure of the glomerulus (G) and evident intertubular congestion (arrows). (3b)** deeper cortex with dilated proximal (P) and distal (D) convoluted tubules (arrows).

**Figure 8:**
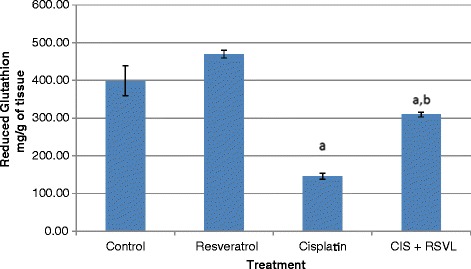
**Effect of CIS and/or RSVL on reduced glutathione (GSH) level in rat kidney homogenate.** Each value presented as mean ± S.D of six rats. ^**a**^Significantly different from control at P > 0.05. ^**b**^Significantly different from CIS at P > 0.05.

Treatment of rats with CIS (7.5 mg/kg,i.p.) caused a significant 3 and 5 fold increase in serum creatinine and blood urea nitrogen, respectively. Simultaneous treatment with RSVL (25 mg/kg,i.p.) reduced the adverse effect of CIS by about 2 and 1.3 fold, respectively (Table 3). Light microscopic examination of kidney section of albino rats after a single dose of CIS showed cortex with shrunken capillary tuft of the glomerulus and widening of the subcapsular space (double head arrow) with deposition of homogenous material (casts) in their lumen (star) and intertubuluar congestion (white arrow) (Figure 4). Concomitant administration of RSVL with CIS showed cortex with normal glomerulus (G), dilated tubules (black arrow) and flaking of cells into tubular lumen (Figure 5). RSVL alone showed a general architecture almost similar to control (Figures 6 and 7). Figure 8 show effect of CIS and/or RSVL on reduced glutathione (GSH) activity in rat kidney homogenate. CIS (7.5 mg/kg) treatment showed a significant decrease in level of GSH by 63.4% compared to control. While, in presence of RSVL (25 mg\kg), the GSH level increased.

**Table 3:** **Effect of CIS and/or RSVL on the serum level of Creatinine and blood urea nitrogen in rats**

**Treatment**	**Creatinine (μmol/L)**	**BUN (mmol/L)**
**Control**	32 ± 2.92	2.12 ± 0.72
**RSVL (25 mg/kg)**	35 ± 1.58	3 ^**b**^ ± 0.73
**CIS (7.5 mg/kg)**	93 ^**a**^ ± 10.32	11 ^**a**^ ± 0.79
**CIS (7.5 mg/kg) + RSVL (25 mg/kg)**	45 ^**a,b**^ ± 2.55	8 ^**a,b**^ ± 1.16

Data are presented as mean ± S.D of five rats.

^**a**^Significantly different from control at P > 0.05. ^**b**^Significantly different from CIS at P > 0.05.

## Discussion

Cisplatin continues to be widely used as a main cancer drug due to its disparity antitumor activity when compared with the other platinum analogues. In tumors and other dividing cells, CIS-DNA intrastrand crosslinks result in cytotoxicity [10].

Recent studies have discovered new protocols, compounds, enzymes and molecular alterations that reduced the side effect of anticancer drugs and enhanced their cytotoxic effects [11-13].

In an attempt to minimize CIS side effects and at the same time increasing its anticancer efficacy, we investigated whether RSVL which is a naturally occurring compound that is found in many food like grapes, can enhance the cytotoxic activity of CIS against the growth of EAC cells *in vivo* and the possible protective role against CIS-induce nephrotoxicity.

In the current study treatment of tumor bearing mice with RSVL (25 mg/kg) enhanced the cytotoxic activity of CIS against the growth of EAC cells with 2.92 folds increase in long-term survivor. It seems that RSVL did not show anti-tumor activity by its own, but rather increased the anti-tumor activity of CIS against the growth of EAC cells (Figure 1 and Table 1)). Combining resveratrol with CIS is a novel strategy that has the potential for improving the antineoplastic activity of CIS [14]. It has been reported that a greatest synergism was observed when resveratrol was administered first followed by the platinum drug (CIS or oxaliplatin) 2 hr later [15]. High cellular level of CIS concentrations in EAC cells has been observed when RSVL was concomitantly administered with CIS (Table 2).This is in a good agreement with previous work in our laboratory, when human breast cancer cell line MCF-7 was treated with simultaneous combination of RSVL and anti-cancer drug DOX resulted in more DOX cellular uptake [5]. The increase in CIS cellular uptake inside Ehrlich cells may be explained by inhibition of P-gp that plays very important role in the absorption, distribution, and elimination of anticancer drug and thus determine its efficacy and toxicity. There were many reports showed that the inhibition of P-gp considered as one of the mechanism of RSVL chemosensitizing effect to anticancer drugs [16,17].

On contrary to our work, yang *et al.,* [18] reported that RSVL enhanced the activity of P-gp in colon cancer cell line. These conflicting findings could be explained on the following basis: MDR can be acquired after initial exposure to the anticancer drugs [19]. In addition several studies have found that some of the well-known P-gp antagonists such as verapamil and cyclosporine A can induce P-gp expression in colon carcinoma cells [20]. So, it is important to know that the time needed for expression and inhibition of P-gp by their antagonists is controversial.

It is well known that DNA damage caused by different cytotoxic agents, induce cell cycle arrest at G_0_-G_1_, S and G_2_-M phases, thereby preventing replication of damaged DNA or aberrant mitosis which if not repaired, may result in either tumorigenesis or apoptosis [21,22].

In our work, simultaneous treatment of CIS with RSVL significantly accumulated EAC cells in sub-G_1_phase and arrested the cells in G_0_/G_1_chek point. The arrest of cells in S phase significantly decreased compared with treatment with CIS alone, which means more cells left the cycle after G_0_/G_1_ check point or S phase and entered the apoptotic sub-G_1_ phase. These result are in agreement with others that investigated the role of RSVL in modulating CIS cytotoxicity by enhanced growth inhibition which may be due to ability of RSVL to induce apoptotic reaction through activation of proapoptotic family members, which works together with cytotoxic effect of chemotherapy agents [13].

Moreover, cell cycle analysis showed that treatment with RSVL plus CIS induced a significantly decrease in the proliferation index (S phase + G_2_/M phase) compared with cells treated with CIS alone, with maximum decrease after 24 hours. This agrees with the uptake study where cisplatin uptake in tumor cells was increased after addition of RSVL with maximum level observed after 24 hours (Table 2). It has been known that the nephrotoxicity of CIS limits the usefulness of this important chemotherapeutic agent [23,24].

Research into the mechanism of CIS nephrotoxicity is an important step for renal protection. One theory involve binding of CIS to sulfhydryl (SH) groups in the kidneys are necessary for enzyme function and depletion of intracellular glutathione lead to renal damage [25]. In agreement with that work our study showed that RSVL treatment inhibited CIS-induced depletion in GSH and protected against CIS induced nephrotoxicity that manifested by restoration of CREA. and BUN to normal level after CIS and RSVL treatment.

It is well known that CIS can cause oxidative stress by suppress cellular antioxidants defenses due to its binding to nucleophilic molecules such as glutathione, methionine or cysteine-rich proteins [26]. At the same time RSVL had ability to suppress of oxidative stress so could prevent CIS induced organ toxicity [13,27].

Light microscopic study confirmed the biochemical findings, where the kidney in rat treated with RSVL and CIS simultaneously showed the cortex with normal glomerulus. Also, the electron microscopic study (data not shown) confirmed these results where simultaneous treatment of CIS with RSVL showed organized apical microvilli, mitochondria and spaces between the cytoplasmic infolding appear within normal and nuclei appear euchromatic.

In conclusion, we demonstrated that RSVL treatment significantly has a potential role in enhancing the antitumor activity of CIS with renal protective effect against its nephrotoxicity.
