# Correction to: Chemosensitizing and nephroprotective effect of resveratrol in cisplatin-treated animals

**DOI:** 10.1186/s12935-019-0750-0

**Published:** 2019-02-12

**Authors:** Abdel-Moneim M. Osman, Suad A. Telity, Zoheir A. Damanhouri, Sameer E. Al-Harthy, Huda M. Al-Kreathy, Wafaa S. Ramadan, Mohamed F. Elshal, Lateef M. Khan, Fatemah Kamel

**Affiliations:** 1 Pharmacology Department, Faculty of Medicine, King Abdulaziz University, P.O. Box 80205, Jeddah, Saudi Arabia; 2 Pharmacology Unit, National Cancer, Institute, Cairo University, Cairo, 11796 Egypt; 3 Anatomy Department, Faculty of Medicine, King Abdul Aziz University, P.O. Box 80205, Jeddah, Saudi Arabia; 4 Department of Biochemistry, Faculty of Science, King Abdulaziz University, P.O. Box 80203, Jeddah, Saudi Arabia; 5 Molecular Biology and Genetic Engineering and Biotechnology Department, Minoufia University, Minoufia, Egypt

## Correction to: Cancer Cell International (2015) 15:6 10.1186/s12935-014-0152-2

Following publication of the original article [1], the authors notified us of an error in the spelling of second author’s name. The correct name spelling is **Suad** A. Telity.
